# The role of high-mobility group box-1 (HMGB-1) in the management of suspected acute appendicitis: useful diagnostic biomarker or just another blind alley?

**DOI:** 10.1186/1757-7241-19-28

**Published:** 2011-04-20

**Authors:** Kjetil Søreide

**Affiliations:** 1 Department of Surgery, Stavanger University Hospital, Stavanger, Norway

## Abstract

Acute abdominal pain is one of the most frequent reasons for admitting patients to the emergency department for surgical evaluation. A wide number of differential diagnoses are available and their pre-test likelihood ratio varies according to the patients' age, gender, duration of symptoms and overall clinical context. While many patients with abdominal pain do not need to be admitted to the hospital wards and even fewer need eventual surgical intervention, the diagnosis of acute appendicitis remains one of the most frequently entertained differential in patients with abdominal pain. In fact, surgery for appendicitis is one of the most frequently performed operations in the Western world. As the authors of the current study point out, the high mobility group box-1 protein (HMGB1) has been known for many years. The study demonstrates in a small pilot that there is a difference in expression of HMGB1 between those with and those without appendicitis. However, is this difference clinically important? Clinically relevant results can only be documented through larger studies comparing its use and expression levels in both healthy subjects, subjects with abdominal pain for other reasons, patients with 'clear-cut' (histopathologically confirmed) appendicitis and in the difficult subgroup of patients with suspected appendicitis and equivocal symptoms.

## 

Acute abdominal pain is one of the most frequent reasons for admitting patients to the emergency department for surgical evaluation. A wide number of differential diagnoses are available and their pre-test likelihood ratio varies according to the patients' age, gender, duration of symptoms and overall clinical context. While many patients with abdominal pain do not need to be admitted to the hospital wards and even fewer need eventual surgical intervention, the diagnosis of acute appendicitis remains one of the most frequently entertained in abdominal pain. In fact, surgery for appendicitis is one of the most frequently performed operations in the Western world. Even today, with current advances in diagnostic imaging and the ever increasing use of laparoscopy, the patient with 'suspected appendicitis' represents a diagnostic challenge. Indeed, early diagnosis remains the most important clinical goal in patients with suspected appendicitis. Large scale studies have demonstrated that while the rates of 'negative' (or 'unnecessary') appendectomies do decline, the rates of perforation remains fairly constant at about 15% [1]. Perforated appendicitis represents a major disease burden for both patient and society, and comes with added morbidity and complications. While appendicitis is not as dreaded now as it was a century ago, mortality is still reported in about 1% of patients. Risk factors are not completely understood more than 100 years after its first description [2]. The diagnosis is still based on clinical examination, optional imaging studies and blood laboratory tests [3,4]. To the latter category belongs white blood cell counts (WBC) and C-reactive protein (CRP) as two of the most frequently evaluated blood test, yet none of them are confirmative for appendicitis as they may either be elevated, within a normal range or associated with other diseases.

Thus, the current study published in the SJTREM by Albayrak et al [5] is important for a number of reasons. For one, the search for new and better diagnostic biomarkers would potentially have great impact on work-up and use of diagnostic imaging if accurate and predictive of disease. In particular this is useful in patients whose clinical symptoms are equivocal. Second, genomic and proteomic biomarkers may shed new light on disease processes needed to discern differences in aetiology and pathogenesis which may eventually help us understand this disease better. Finally, a drive towards non-operative management of "non-complicated" appendicitis has been advocated through randomised controlled trials recently [6]. However, of concern is the fact that at current diagnostic tools are non-specific so one cannot at present reliably confirm that appendicitis is the true entity that is being treated in such trials. Consequently, the results are not generalisable and not immediately valid nor advisable for use in the general population at large [7,8].

As the authors of the current study point out [5], the high mobility group box-1 protein (HMGB1) has been known for many years as a nuclear chromosomal protein. Its role as a pro-inflammatory cytokine in sepsis and rheumatoid arthritis has been described, and more recently its role in community-acquired infections and sepsis investigated [9].

HMGB1 is an intracellular protein that can translocate to the nucleus where it binds DNA and regulates gene expression. It can also be released from cells, in which extracellular form it can bind to an inflammatory receptor called Receptor for Advanced Glycan Endproducts (RAGE). Activated macrophages and monocytes secrete HMGB1 as a cytokine in inflammation. The mechanism of inflammation and damage is binding to toll-like receptor 4 (TLR4), which mediates HMGB1-dependent activation of macrophage cytokine release. This positions HMGB1 at the intersection of sterile and infectious inflammatory responses. Thus, the increased level of HMGB1 likely reflects a systemic inflammatory response syndrome (SIRS) in patients with appendicitis, that may resemble the same or similar mechanisms as previously detailed for trauma patients and following post-injury events [10-13]. However, the jump from molecular mechanisms that may be a central player, or just a bystander effect, of the primary insult, is a relatively premature closure. As Stahel and colleagues highlighted for mechanisms explored in injury [10], the metabolic effects are characterized by a network of interactions, and cross-linkage and cross-over effects of which it is extremely hard if not possible to predict an outcome based on one sole player amongst the mingling molecules.

The diagnostic value of HMGB1 levels was investigated using ROC curve analysis in this study by Albayrak et al [5]. The use of ROC analysis is an appropriate method in evaluation for various biomarkers [14,15]. The curve shows that a high discriminative ability was not obtained, although levels between diseased patients and controls differed significantly. For the diagnosis of acute appendicitis, the best cut-off point for HMGB1 was at 25 ng/ml. The calculated sensitivity, specificity, positive predictive value and negative predictive value were calculated as 72%, 73%, 88% and 45%, respectively (area under curve = 0.781), which is not comparably better to the accuracy of WBC or CRP already in clinical use [16]. So it appears, as testing for HMBG1 is not readily available 24-7-365 in most clinical chemistry labs, nor demonstrable cheaper or more cost-efficient than other available tests, it will not replace a standard work-up panel as of yet.

Nonetheless, the current study demonstrates that there is a difference in expression of HMGB1 between those with and those without appendicitis. Whether the question under investigation will give clinically important answers can only be addressed through future, larger studies comparing the use and expression levels of HMGB1 in both healthy subjects, subjects with abdominal pain for other reasons, patients with 'clear-cut' (histopathologically confirmed) appendicitis and in the difficult subgroup of patients with suspected appendicitis and equivocal symptoms. Whether this may truly prove a useful diagnostic biomarker among the increasing number of alternatives investigated [17-19], or merely be yet another blind alley in the surge for the correct diagnosis of acute appendicitis remains to be seen.
